# Sustainable Food Systems: The Case of Functional Compounds towards the Development of Clean Label Food Products

**DOI:** 10.3390/foods11182796

**Published:** 2022-09-10

**Authors:** Maria Alexandri, Vasiliki Kachrimanidou, Harris Papapostolou, Aikaterini Papadaki, Nikolaos Kopsahelis

**Affiliations:** Department of Food Science and Technology, Ionian University, 28100 Argostoli, Kefalonia, Greece

**Keywords:** functional compounds, clean label foods, phenolics, carotenoids, pectins, oligosaccharides, bioactive peptides, sustainable food systems

## Abstract

The addition of natural components with functional properties in novel food formulations confers one of the main challenges that the modern food industry is called to face. New EU directives and the global turn to circular economy models are also pressing the agro-industrial sector to adopt cradle-to-cradle approaches for their by-products and waste streams. This review aims to present the concept of “sustainable functional compounds”, emphasizing on some main bioactive compounds that could be recovered or biotechnologically produced from renewable resources. Herein, and in view of their efficient and “greener” production and extraction, emerging technologies, together with their possible advantages or drawbacks, are presented and discussed. Μodern examples of novel, clean label food products that are composed of sustainable functional compounds are summarized. Finally, some action plans towards the establishment of sustainable food systems are suggested.

## 1. Introduction

Waste generation and the efficient handling of it constitutes an undeniable problem of the modern world. The food sector produces tremendous amounts of waste which can no longer be disregarded, and strict measures for both its reduction and efficient handling have been proposed. The impact of food waste on the environment is immense as about 8–10% of greenhouse gas emissions are attributed to unconsumed food [1]. To this end, the Food and Agriculture Organization of the United Nations (FAO) initiated the Food Index Report, which is in line with the Sustainable Development Goal 12.3 (SDG 12.3). SDG 12.3 is committed to reducing food waste by 50%, taking into account the losses derived from the entire production line (e.g., field, supply chains, etc.) to the consumer level [2]. The Food Loss Index records food waste occurring worldwide, accounting losses derived from retail, from food services but also from households [1].

According to the U.S. Environmental Protection Agency, in 2020 the estimated food waste per capita accounted for 139 kg, from which 46% was generated from food services and 42% was generated from households. In Europe, the numbers differ significantly among the countries, however, approximately 73 kg of food from the European households is wasted per capita [1]. This waste include both the edible and inedible parts of food, whereas the survey concludes that the amount of household waste is comparable among the countries, regardless of the corresponding income category. Overall, the latest report that was published from Food Waste Index estimated that the total global food waste for 2019 accounted for 931 million tonnes, of which households distributed 61% of it, 26% came from food services and 13% came from the retail sector [1].

The implementation of the aforementioned goals could be partially achieved via the dynamic exploitation of these waste and/or by-product streams. The largest impact of these streams could be realized via their fractionation into value-added components and their bioconversion into products of commercial interest [3]. A complete exhaust stream could then be disposed of, with fewer environmental consequences. As a matter of fact, such strategies could ensure resource efficiency in compliance with those of the bioeconomy era.

Likewise, these policies have raised environmental awareness and created a tendency towards a healthier and more sustainable lifestyle. The modern food industry is therefore forced by society to conform to the new reality and develop efficient waste management outlooks. A great number of review articles deal with the valuable components that can be recovered by food and agricultural waste, especially from fruits and vegetables [4,5,6]. These compounds include mainly antioxidants (phenolic compounds and carotenoids) as well as polysaccharides, proteins, fibers, and fats, which can be channeled back to the production chain for the development of novel, healthier and more sustainable foods. Meals of various seeds such as flaxseed and chia seed are also sources of bioactive compounds and their introduction into the value chain could aid the development of novel, functional foods [7,8]. For instance, the inclusion of flaxseed in the diet of laying hens has led to the production of eggs rich in polyunsaturated fatty acids [7,9], whereas feeding broilers with rations enriched with linseed oil resulted in eggs with increased ratio of polyunsaturated fatty acids against monounsaturated fatty acids [10].

Biomass bioconversion to value-added compounds constitutes another major valorization strategy. Microorganisms produce valuable products via their metabolism, assimilating various carbon sources derived from such waste and by-product streams. Organic acids, pigments, oligosaccharides and lipids are some examples of such metabolites with potential food applications [11]. Microalgae confer another source of valuable compounds and their incorporation in animal feeds had led to the production of meat with higher omega-3 content and antioxidant capacity [12]. Some microorganisms (e.g., lactobacilli, bifidobacteria, etc.) constitute well-known probiotics, as they promote physiological gut functioning, among other health benefits [13]. This practice ensures resource efficiency and meets the consumers’ demand for healthier products, as is signified by the thriving demand for “functional foods”.

Numerous studies advocate that diets that are rich in functional compounds could be advantageous towards human health. Antioxidants (mainly phenolic compounds, carotenoids and other phytochemicals) are the most studied functional compounds, considering their recent inclusion (in 2017) in the prebiotic definition [14]. Many studies corroborate their health-promoting properties, discussing that they display not only antioxidant activities, but also anti-inflammatory, antimicrobial and anticarcinogenic properties [15]. Their consumption could possibly decrease the risk of various chronic diseases such as cardiovascular disease and cancer [16]. Some fatty acids, especially the ones belonging to the omega-3 polyunsaturated fatty acids (n-3 PUFA), are another category of bioactive compound with a huge scientific interest in them. The main representatives of this class of compounds include α-linolenic acid (ALA, 18:3 n-3), docosahexanoic acid (DHA, 22:6 n-3) and eicosapentaenoic acid (EPA, 20:5 n-3). The consumption of foods that are rich in n-3 PUFA is highly recommended as these compounds present a beneficial effect towards cardiovascular diseases and some forms of cancer [17].

The sustainable incorporation of these bioactive compounds in food and beverages requires their equally sustainable recovery from waste and by-product streams. Novel and “green” extraction methods have been developed targeting, the effective and economical recovery of these compounds. Supercritical fluid extraction (SFE), ultrasound-assisted extraction (UAE), microwave-assisted extraction (MAE), enzymatic assisted extraction (EAE), pulsed electric fields (PEF) and natural deep eutectic solvents (NADES) are the most studied extraction techniques [15]. However, each method possesses advantages and limitations and is highly affected by the raw materials and the target compound that is to be extracted.

This review aims to provide an outlook of the main categories of functional/bioactive compounds that can be recovered or biotechnologically produced from agro-food waste streams. Green and sustainable extraction and production methods are presented and discussed. Finally, the current application trends of these “sustainable bioactive components” for the development of novel and functional foods are summarized. This unified outlook could help researchers to identify the gaps into the sustainable incorporation of these valuable compounds in everyday life. At the same time, this work highlights that integrated sustainable food systems could be established only through an organized action plan which will include not only green extraction processes and biotechnological approaches, but also policies and the successful production of “clean label food products” with proper features for human consumption.

## 2. Functional Compounds: Definition, Importance and Trends

The term functional or bioactive compound is used to denote a class of diverse molecules that possess biological activity. These compounds occur naturally as plant and/or microbial metabolites. They are part of the food chain, eliciting essential or non-essential properties [13,16]. The interest for functional compounds is constantly flourishing as an equally increasing number of studies proves or highlights their benefits to human health. These compounds are the tangible example of Hippocrates’ saying “Let food be your medicine”, as they manifest a plethora of health-promoting and -protective properties such as antioxidant activity, anti-inflammatory, antifungal, antimicrobial and antiviral, to name a few [18].

Foods containing bioactive compounds with recognizable health-promoting properties can be characterized as functional. The properties indicating that a component possesses a certain bioactivity include the management, prevention and treatment of diseases through triggering or manipulating certain mechanisms in the human organism [18]. Even though an official definition of functional foods has yet to be provided, in 2017, the Functional Food Center gave the following definition, “Natural or processed foods that contain known or unknown biologically-active compounds; which, in defined, effective, and non-toxic amounts, provide a clinically proven and documented health benefit for the prevention, management, or treatment of chronic diseases” [18]. Functional foods’ market accounted for approximately 178 billion U.S. dollars in 2019, while this is estimated to reach 268 billion U.S. dollars by 2027 [19]. The market includes vitamins and minerals, dietary fibers, probiotics, prebiotics, proteins and amino acids.

Bioactive compounds are considered to be keystones for functional food development. As functional compounds are quite heterogeneous, and new molecules with biological activity are constantly being discovered, this review will focus on organic acids, phytochemicals (mainly phenolic compounds and carotenoids), oligosaccharides, pectins and bioactive peptides.

### 2.1. Organic Acids

The addition of organic acids in food and feed represents a long-established practice, which is mainly followed to increase the shelf-life of the end-product. Lactic acid, citric acid, benzoic acid, propionic acid, sorbic acid, fumaric acid, formic and succinic acid are the mostly used organic acids in various food applications [20]. The common mode of action of all these organic acids lies in their ability to penetrate the microbial cell wall, thus exhibiting an antimicrobial activity. The addition of organic acids in food can reduce the risk of infection from various bacteria such as *E.coli*, *Clostridia* spp., *Listeria* spp., *Salmonella* and *Coliforms* spp. [20]. Moreover, organic acids act as acidifiers, regulating the growth of specific pathogenic bacteria in the human gut [21]. Another application of organic acids is their utilization in feed formulations for broilers and pigs as substitutes for antibiotic growth promoters [22]. Many studies indicate that the inclusion of organic acids in the diet of certain animals enhances their gut health, minimizing the need for antibiotics, hence reducing the risk of antibiotic resistance [23,24,25]. The addition of organic acids in poultry feed increases their growth and feed productivity and also the digestibility of the nutritious components [26]. Owing to all these positive properties, these organic acids are also characterized as dietary organic acids [26]. Recently, Ma et al. [27] showed the positive effect of mixed organic acids (formic, propionic, lactic, acetic, malic and citric acids) on antioxidant properties, enzymatic activity and the intestinal health in broilers.

Most of the organic acids are mainly produced via microbial pathways and to a lesser extent, through petrochemicals routes. Yields and productivities vary greatly as they highly depend on the strain and the fermentation substrate employed. Characteristic examples of widely produced organic acids by fermentation are lactic, acetic, and citric acids. Lactic acid is maybe one of the most interesting organic acids, finding applications not only in food and feed but also in cosmetics, pharmaceuticals and also as a monomer for the synthesis of poly(lactic acid) (PLA), a biodegradable polymer [21,28]. Lactic acid bacteria (LAB) such as *Lactobacillus*, *Pediococcus*, *Leuconostoc*, *Lactococcus*, and *Streptococcus* are the most well-known lactic acid producers. These strains are regularly utilized as starter cultures for the production of various fermented products such as milk, meat and vegetables [21]. Acetic acid presents a higher antimicrobial effect when compared to lactic acid due to its higher pKa (4.75). It is the main acid of vinegar, presenting well-known health benefits upon consumption.

Succinic acid is widely used in the food industry as an anti-microbial agent, an acidulant and as a flavoring agent. Among the organic acids of interest, the predominant production of succinic acid is still based on chemical processes. However, the number of studies elaborating on its biotechnological production from renewable resources is constantly increasing. Succinic acid can be produced with the use of various wild type strains through the TCA cycle. These strains can belong to LAB or to various strains that are isolated from the bovine rumen such as *Actinobacillus succinogenes* and *Basfia succiniciproducens*. Citric acid is traditionally produced by microbial fermentation, with the fungal strain *Aspergillus niger* being the industrially utilized one [29]. Owing to its antioxidant and acidifying properties, citric acid is added into food and beverages to prevent the oxidation of sensitive components and as a flavor amplifier [29]. In all cases, strain improvement and the selection of appropriate substrates are critical factors for efficient and economically viable bioprocesses.

### 2.2. Phenolic Compounds and Other Antioxidants

Phenolic compounds are the most recognized functional compounds in food and beverages. They comprise plant secondary metabolites, which are widely distributed among vegetables, cereals and fruits [30]. The biosynthesis of phenolic compounds in plants is carried out mainly through the shikimic pathway, from which compounds with a different carbon skeleton can be produced. The main categories include phenolic acids, flavonoids, stilbenes, tannins, lignans, coumarins, curcuminoids and quinones. Their biological role is to assist the plant’s growth and its reproduction and to offer it protection against pathogens and ultraviolet radiation [30]. A great number of studies highlight the need to include phenolic-rich foods in human’s diet, as their consumption has been positively correlated with the reduced risk of plenty of chronic diseases such as different cancer types, cardiovascular diseases, and diabetes, to name a few [4,5,6,13]. Phenolics owe their antioxidant activity to their ability to donate hydrogen and therefore stop free radical production. Free radicals are responsible for cell destruction, premature aging and the development of some diseases such as cancer and Alzheimer’s disease [5]. Phenolic compounds also present antimicrobial activity against a variety of pathogens, inhibiting their growth and thus increasing the shelf-life of foodstuffs [30]. The most abundant phenolic compounds are phenolic acids and flavonoids, which can be found in considerable amounts in many plant-based foods. Gallic and ellagic acids are the most common phenolic acids, whereas caffeic and ferulic acids are the prevalent hydroxycinnamic acids in many foods. Among flavonoids, catechin and epicatechin belong to the most common flavan-3-ols that are found in many fruits and vegetables [31].

Carotenoids constitute another category of secondary plant and microbial metabolites with a strong bioactive activity and a broad color spectrum. To date, more than 1100 carotenoids have been identified, and they have been extensively reviewed regarding their health benefits, recovery methods and production methods [32,33,34]. The inability of the human body to synthesize carotenoids renders them essential for the human diet, as many carotenoids act as precursors for the synthesis of vitamins (e.g., pro-vitamin A) [32]. The most abundant carotenoids that are found in nature are β-carotene and lycopene, followed by zeaxanthin, β-cryptoxanthin, α-carotene and lutein [34], while astaxanthin is the predominant carotenoid that is found in various marine organisms [33]. Carotenoids applications extend from food to cosmetics and from cosmetics to pharmaceuticals, as their unique structure permits their inclusion in many biological systems. Similar to phenolic compounds, carotenoids elicit a key role in scavenging ROS, thus reducing the risk of oxidative stress and cell damages. Epidemiological, preclinical and clinical studies have been already conducted, or are still on-going, regarding the positive effect of carotenoids’ consumption on obesity, cancer, cardiovascular diseases, type 2 diabetes, eye health and many other chronic diseases [33].

Even though the technology for the chemical synthesis is inexpensive and efficient, synthetic carotenoids have been accused of causing various health implications. The rate of their biotechnological production is now facing an increase, as new strains demonstrating a high capacity for producing carotenoids have been discovered, and the production technology is constantly evolving. Carotenoids could also be extracted from plants, however the process is expensive and susceptible to environmental and geographical restrictions [33,34].

### 2.3. Oligosaccharides

Oligosaccharides are a functional group that encompasses a wide range of compounds: from simple carbohydrates to soluble fibers and with different degrees of polymerization (DP) [35]. They are categorized according to their sugar monomers, and the most well-known functional oligosaccharides are fructooligosaccharides (FOS), xylooligosaccharides (XOS), galactooligosaccharides (GOS), iso-maltooligosaccharides (IMOS) and mannan oligosaccharides [36]. FOS and GOS are broadly employed in the food industry due to their advanced technological properties (e.g., water-holding capacity and gel-forming ability) and the established health-promoting benefits that they have on gut microbiota [35]. They naturally occur in many foods such as fruits and vegetables, honey, milk, and lentils [36]. Similar to FOS and GOS, XOS and pectic oligosaccharides are also gaining attention. XOS and pectic oligosaccharides can be produced from inexpensive renewable resources, which considerably lowers their production cost [35]. There are many studies that have investigated the inexpensive and sustainable production schemes for both XOS and pectic oligosaccharides [35,36,37].

Their conventional applications focus on the partial replacement of sugar and fat or they act as fortifying agents [35,38]. However, their emerging applications are mainly associated with their prebiotic potential. The term “prebiotic” denotes any food component that is non-digestible in the human intestine, and that enhances the growth of the gut microbiome with beneficial effects on human health [35]. These types of oligosaccharides promote the growth of certain microorganisms that are responsible for the production of short chain fatty acids (e.g., acetate, propionate, butyrate, lactate) which are associated with a reduced risk of inflammation, hypercholesterolemia, colorectal cancer, obesity, type 2 diabetes and other diseases [35].

### 2.4. Pectins

Pectins are a highly heterogeneous category of functional compound. From a chemical point of view, pectins are complex polysaccharides composed of homogalacturonans (HG), rhamnogalacturonan-I (RG-I), rhamnogalacturonan-II (RG-II) and smaller portions of xylogalacturonan and apiogalacturonan moieties [39,40]. Their main skeleton consists of repeated units of α-D-galacturonic acid, linked with 1,4-glucosidic bonds, and esterified with methyl-residues at C-6 [40]. The diversity in their structure results from the variety that occurs due to different side chains or the extent of cross-linking, and the location of the *O*-acetyl, methyl ester groups and other side groups, and the presence of rhamnosyl moieties in the backbone moieties. Their natural occurrence is in the plant’s middle lamella as well as in both primary and secondary cell walls [39]. The production of pectins is commercially carried out via extraction from various plant sources (mainly fruits and vegetables and their corresponding by-products), and applications are found not only in the food industry, but also in cosmetics and pharmaceuticals. These multipurpose compounds present stabilizing and thickening properties and they can also act as emulsifying and gelling agents [39]. European regulation dictates that commercial pectins should contain at least 65% α-D-galacturonic acid [39]. Commercial pectin products can also be further categorized into high- and low-methoxy pectin, depending on the degree of methyl esterification (DM) (higher or lower than 50%, respectively). The extraction method greatly affects the pectin’s structure that consequently determines its end applications. The DM plays a critical role in the properties, hence it dictates the end applications of the produced pectins. Eighty-five and a half percent of all commercial pectins are traditionally derived from citrus peel, followed by apple pomace (14%) and the by-products of juice production, even though alternative sources are currently being investigated [40].

Besides their technological and mechanical properties, pectins also present important health benefits such as antioxidant activities, anticancer, anti-diabetic and anti-inflammatory effects, to name a few. Fruit pectins exhibit prebiotic properties as they are indigestible, but still fermentable by the gut microbiota [39]. Their inclusion in food formulations has paved the way for a new emerging category of functional foods. To this end, the sustainable extraction of pectin and its production methods are in the spotlight of many research groups.

### 2.5. Bioactive Peptides

The nutritional value and biological role of proteins is known; however, proteins and protein-based compounds possess many more functional properties rather than just providing energy to the cell [41]. Bioactive peptides are the products of protein hydrolysis, and consist of 2–20 amino acids, thereby exhibiting remarkable health effects [42]. These functional compounds can be obtained via enzymatic hydrolysis and microbial fermentation. There are a great number of review and research articles that have been published in the literature dealing with novel bioactive peptides isolated from different sources, such as food-based ones [41,43], from dairy products [44], from cereal-based foods such as amaranth, quinoa and chia [42,45], those of microbial origin [46], and those that are plant-based such as those from hemp [47].

The spectrum of health benefits that they provide is wide, including anticancer properties, anti-diabetic, antioxidant, hypocholesterolemic and neuroprotective properties, among others [41,42,48]. Clinical evidence is still being collected on the effect of dietary bioactive peptides on the gastrointestinal tract (GIT), but a positive correlation between the consumption of these functional compounds and gastrointestinal homeostasis seems to exist [49]. Possible toxicity implications, digestibility, consumer’s acceptability and the manipulation of the produced peptides to present the desirable properties are the main fields of research on this topic [42].

## 3. Chemical/Conventional Production of Functional Compounds

**Figure 1:**
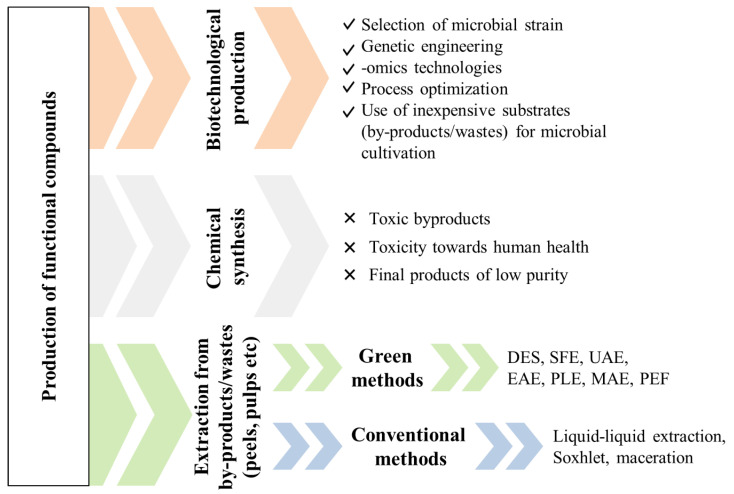
Conventional and emerging techniques for the recovery and production of functional compounds.

Traditionally, chemical compounds with specific functional properties could be either extracted from plant matrices or produced via chemical synthesis (Figure 1). The conventional extraction methods of phenolic compounds from plant matrices include solvent extraction, Soxhlet extraction, maceration, decoction and hydro-distillation [50]. The main principle involves the use of an adequate organic solvent and the application of high temperatures for a specific amount of time to recover the target compounds. Solvent extraction is the most well-established extraction process for the recovery of phenolic compounds [51]. These methods have been previously reviewed, and their advantages and disadvantages have been extensively discussed [4,15,50,52]. The suitability of the solvent is related to the polarity of the compounds that will be extracted. For example, polar compounds such as phenolics require polar solvents such as ethanol, methanol or ethyl acetate, whereas non-polar ones such as lipids are extracted with non-polar solvents such as hexane and petroleum ether. Another parameter affecting the solvent selection is the cost and toxicity, especially when the extracted compounds are intended for food or pharmaceutical applications.

Solvent extraction is also employed for carotenoids extraction from plants or from marine organisms [32,33,34]. Similar to phenolic compounds, the selection of the most suitable solvent greatly affects the extraction yield of carotenoids. Due to their hydrophobic nature, hexane and petroleum ether are the most common solvents, while acetone and ethyl acetate are preferred for more polar carotenoids. Saponification has also been proposed for carotenoids such as lutein that can be found in esterified forms with fatty acids [32]. The chemical synthesis of carotenoids was introduced in 1950s and it is based on Wittig reactions. Lycopene, β-carotene, astaxanthin and canthaxanthin are some examples of industrially produced carotenoids that are acquired by chemical synthesis [53]. Chemical routes offer stable commercial products at a low cost, however, the latest findings have associated the consumption of synthetic carotenoids with health implications [54]. Likewise, the production of natural carotenoids in a sustainable and cost-effective manner is, nowadays, constantly being developed.

Other bioactive compounds, especially oligosaccharides, pectins and peptides, are extracted via alkaline extraction, mainly with the use of NaOH [40,43,55]. For example, the alkaline extraction of the tea residues from the herb *Apocynum venetum* L. has resulted in polysaccharide conjugates (which are bound to proteins) with high emulsification properties [55]. Heat reflux extraction (HRE) is another process that is frequently used for the extraction of polysaccharides and pectins from plant matrices [56]. However, the high temperatures and extended extraction times that are required could alter the structure of the polysaccharides, thus modifying their functional properties. Alkaline hydrolysis is generally considered to be effective, however the structural alterations that can occur could lead to losses in functionality, particularly in the case of proteins and/or peptides. Physical processes have also been proposed for protein extraction such as colloidal milling and high-speed blending [43].

Another traditional method for pectin extraction from apple and citrus pomaces is acid hydrolysis and alcohol precipitation [40,57]. The extraction yield depends on the acid used (mineral or organic acids) and the extraction temperatures. Hot water extraction has also been suggested as a more environmentally friendly alternative; however, this method results in low pectin yields [40].

Even though all these methods are well established, they present various drawbacks that are mostly related to the high solvent quantities, toxicity, long extraction times and a low technology readiness level (TRL), thus hampering their industrial application. Moreover, the use of conventional solvents and the high electricity consumption required significantly increases the carbon footprint of these processes [58]. Novel, non-conventional techniques are presently being evaluated for the recovery of functional compounds, aspiring to achieve high extraction efficiencies with a low environmental impact.

## 4. Strategies for the Production of Clean and Sustainable Compounds

The transition to the bioeconomy era dictates the complete exploitation of waste and by-product streams. The previous linear model of “take, make and dispose of” is now slowly converted to a more sustainable framework, with resource recycling and recovery at the epicenter. The concept of circular economy created the idea of modern biorefineries, where waste and by-product streams would be treated as “resources” and further valorized either via fractionation or microbial conversion to value-added compounds [59].

The agro-food sector produces large amounts of waste and by-product streams, generated during every link in their long supply chain (field, retail, supply chain and consumer). Some of these wastes (e.g., spoiled food and food at the end of its expiration date, etc.) could be avoided via better handling and the use of adequate societal strategies. Other forms of waste (e.g., fruit peels, seeds, pulps, leaves, branches and pruning residues, etc.) are unavoidable, and therefore they should be further processed within the context of circular economy. Especially, fruit and vegetable residues are rich in many bioactive compounds of interest that could be recovered and re-introduced in the food chain. The apparent simplicity of such a concept hides many obstacles that the modern food industry should overcome. The recovery of these compounds should impose no danger to human health or the environment. Moreover, the processes should have a low carbon footprint and at the same time, a high extraction efficiency and a low cost.

**Figure 2:**
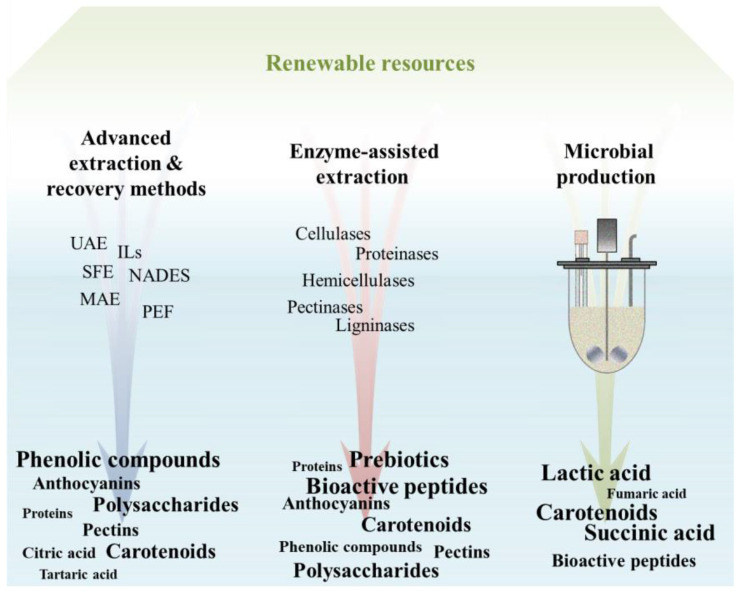
Overview of green and sustainable extraction and production methods of various bioactive compounds.

To this end, green extraction techniques have been developed and researchers are making huge efforts to achieve all of these aforementioned goals. The next section elaborates on green and sustainable production, and extraction techniques are presented and discussed, together with the latest (those which occurred in the last three years) advances in bioactive compounds recovery (Figure 2). These recent advances will shed new light on the development of novel, “clean” label foods, containing zero synthetic components and providing health benefits to the consumers, while their production would present a low environmental impact. The concept of clean label foods is presented in Section 5.

### 4.1. Advanced Extraction and Recovery Methods

Based on Scopus search, more than 100 review articles have been published in the past 10 years regarding green techniques for the recovery of bioactive compounds. This publication number is commensurate with the flourishing interest in novel, green extraction processes, which combine eco-friendly approaches with high extraction yields. The green technologies that have attracted attention the past years include supercritical fluid extraction (SFE), pulsed electric fields (PEF), microwave-assisted extraction (MAE), ultrasound-assisted extraction (UAE), ionic liquids (ILs) and natural deep eutectic solvents (NADES), among others. The principles, advantages and disadvantages of each method are presented in Table 1.

The high diversity and complexity of waste and by-product streams requires process optimization, as different conditions are necessary, depending on the raw materials used and the molecules that are to be extracted. In Table 2, Table 3, Table 4 and Table 5, an overview of the latest studies that have been carried out on the implementation of green extraction techniques for the recovery of different bioactive compounds from waste streams is compiled. It is evident that phenolic compounds and carotenoids monopolize the interest of the researchers (Table 2 and Table 3). UAE, MAE, PEF and PLE extraction using NADES, in combination or individually employed, are the preferred extraction techniques. Many studies evaluate not only various parameters but also compare different extraction methods. To this end, Saldaña et al. [69] investigated the effect of PLE using various concentrations of ethanol (30–100%), water or 5% citric acid, under different temperatures (40–160 °C) and pressures (50 or 200 bar) for the extraction of phenolic compounds from cranberry juice. The authors also compared the PLE results to conventional solvent extraction with 98% acidified methanol. Aqueous EOH, at temperatures above 120 °C, enhanced the extraction yield of the phenolic compounds, while the maximum recovery was obtained with 30% EOH at a temperature range of 140–160 °C, when operating at 50 bar. Pressurized ethanol presented selectivity toward anthocyanins extraction, however the highest extraction yields were achieved at different conditions to total phenolics, and the result was independent of the temperature employed (Table 2). The PLE method resulted in similar extraction yields to that of the conventional solvent extraction with methanol. Methanol constitutes an efficient extraction solvent, with superior properties to ethanol; however, it presents high toxicity. Likewise, solvent extraction using ethanol leads to, in general, lower recovery yields when compared to that of methanol. Pressurized ethanol is a sustainable alternative that also allows for the sequential extraction of total phenolics (low molecular weight compounds) and anthocyanins (high molecular weight phenolic compounds).

Hashemi et al. [87] evaluated different green extraction methods (UAE, MAE and OHAE) as well as conventional hot water extraction, for the recovery of antioxidants from saffron petals. The extracts obtained by all the novel methods exhibited higher antioxidant activities in comparison to the conventionally recovered ones. Moreover, the OHAE extracts showed a considerable level of antimicrobial activity against the foodborne pathogens of *Klebsiella pneumonia*, *Salmonella typhimurium* and *Staphylococcus aureus*.

Chamika et al. [92] compared the recovery yield of bioactive compounds obtained from different waste parts of sea urchins (*Stomopneustes variolaris*) using SWE or Soxhlet extraction. SWE proved a more efficient method for the recovery of total phenolics, proteins and sugars, while the corresponding extracts exhibited also higher antioxidant, anti-inflammatory, antidiabetic, and anti-cancer properties than the ones obtained by the conventional methods. However, the authors highlighted the possible negative effect of the high temperatures (110–230 °C) employed to the final extracts’ overall quality. Symes et al. [72] evaluated the extraction of antioxidants from asparagus roots using either ILs or PEF or conventional solvent extraction. Their results indicated that the extraction capacity of ILs was 70 times higher in comparison to that of PEF.

The extraction of natural dyes from renewable resources has been also a matter of intense investigation (Table 3). Astaxanthin, lycopene, β-carotene and anthocyanins are the most studied pigments in that field [6,93]. Anthocyanins, lycopene and β-carotene can be recovered from fruit and vegetable waste and by-product streams [6]. Astaxanthin constitutes an important carotenoid, exhibiting higher antioxidant activities than both β-carotene and α-tocophenol do [94]. Carotenoids are heat- and light-sensitive and they are easily oxidized compounds, therefore the selected green extraction method should recover them at low temperatures and after a short extraction time. Such methods are presented in Table 1. UAE requires lower energy to achieve similar extraction efficiencies to that of MAW, suggesting that UAE is probably a more favorable extraction method, at least as far as the carotenoids are concerned [95].

**Table 3:** Recent advances regarding the recovery of various carotenoids from waste and by-product streams.

Raw Material	Method	Results	Reference
Crustacean waste streams	MW pretreatment with EOH at 140 °C, 300 W for 90 s and subsequent SFE extraction at 500 bar, 40 °C, 13 wt% EOH as co-solvent	Astaxanthin extraction yield 4.44 ± 0.41 μg/g dry residues	[94]
Passion fruit peels	Olive oil in combination with UAE at 47 °C, 100 W, for 39 min, and solid-to-oil ratio 1–30 g/100 mL	Carotenoids: 1241.95 μg/100 g peels (extraction efficiency of 91.4%)	[95]
Carrot juice processing waste	Flaxseed oil in combination with MAE at 165 W, for 9.39 min, solid-to-oil ratio 1:8.06 g/g	Carotenoids’ extraction efficiency 77.48%	[96]
Tomato processing by-products	PEF with 10 g sample at 5 kV/cm and 5 kJ/Kg and then extracted with ethyl lactate at 1:40 solid-to-liquid ratio	Lycopene: 9930 mg/kg DW	[97]
*Bactris gasipaes* fruit pulp	Aqueous solution of 140 mM of the IL [N_1, 1, 1, 10_]Br at 0.15 solid-to-liquid ratio for 8.2 min	Carotenoids: 88.7 ± 0.9 μg/g dried biomass	[58]
Shrimp waste	ILs-in-water microemulsion of [P_4448_]Br/(TX-100 + n-butanol)/water in combination with UAE (50 W, 60 min)	Astaxanthin extraction efficiency: 32.47 μg/g	[98]
Sunflower petals and florets	NADES composed of D,L-menthol: D,L-lactic acid (1:2), under (0.93:1 *v*/*v* solvent-to-water ratio and 0.15:10 *w*/*v* sunflower:liquid ratio under stirring at 200 rpm for 2 h, at 40 °C after enzymatic pretreatment with Viscozyme (0.58% *v*/*v*)	Carotenoids: 1449 mg/100 g biomass	[99]
Brown crab shell residues	NADES composed of menthol: myristic acid (8:1), NADES-to-waste ratio of 1: 0.25 (*w/w*) under stirring at 60 rpm, temperature 60 °C, 2 h extraction time	Astaxanthin:9.3 ± 0.8 μg/g dry residue	[100]
Shrimp residues	Solvent extraction with DES: 5% (*m/v*) choline chloride: Glycerol, 10 min incubation using UA at 50/60 Hz and 1:10 solid-to-liquid ratio	Total carotenoids: 737.69 μg/g; Astaxanthin content: 32.71 μg/g	[101]
Fermented shrimp waste	CO_2_-SFE with EOH at 300 bar, 60 °C and flow 6 mL/min	Astaxanthin: 0.52 μg/g	[102]
Shrimp wastes	UAE (30 s ON and 30 s OFF pulse sequence interval, 54.43% amplitude) and NADES (choline chloride:lactic acid at molar ratio 1:1.02) and 10% water, 1:10 solid-to-solvent ratio, 39.23 min extraction time	Astaxanthin: 68.98 ± 1.22 μg ASX/g	[103]
Pumpkin pulp	Canola oil as extraction solvent at 1:10 solid-to-oil ratio, at 60 °C, under stirring at 250 rpm and 21.8 min extraction time	Total carotenoids: 373.2 μg β-carotene equivalents/g dried pumpkin pulp	[104]
Beetroot waste	DES composed of chloride hexahydrate and urea (2:1)	Total betalain: 3.99 ± 0.26 mg/g	[105]

An integrated approach that used MW as the pretreatment step before CO_2_-SFE with ethanol as a co-solvent for astaxanthin recovery from crab waste was firstly introduced by Nunes et al. [94]. MW pretreatment disrupted the carotenoprotein complex, facilitating the subsequent SFE extraction. Another promising green extraction process that is mainly directed at carotenoids involves the application of edible vegetable oils as extraction solvents [106]. Vegetable oils are able to extract carotenoids from various sources at high yields without the need of applying highly sophisticated techniques, while (and most importantly) the resulting extract can be directly used for food applications. In addition, by using oils that are rich in valuable components (such as omega-3 fatty acids and tocopherols), the bioactivity of the extracts is additionally enhanced [106]. Sharma et al. [107] studied the effectiveness of UAE and MAE in conjunction to edible vegetable oils (namely corn and olive oil) as alternative green solvents for the recovery of carotenoids and phenolic compounds from sea buckthorn pomace. The highest value of total carotenoids (34.35 mg/100 g of oil extracts) and total phenolic compounds (405.58 mg GAE/g extract) were achieved via UAE using olive oil as green solvent. These values were higher in comparison to those achieved from using a conventional solvent extraction, and they also exhibited enhanced antioxidant activities (93.67% to 58.57%). As shown in Table 3, the extraction of carotenoids from fruits, vegetables or shrimps using edible oils is still under investigation in the literature, mostly in combination with other green technologies.

The purity of carotenoid extracts must be above 94% in order to meet the demands of the food sector. To this end, De Souza Mesquita et al. [58] screened different ILs in ethanolic or aqueous solutions in order to achieve the highest extraction efficiency of carotenoids from *Bactris gasipaes* fruit pulp. Their results indicated that an aqueous solution of the IL [N_1, 1, 1, 10_]Br recovered the highest percentage of carotenoids from the fruit waste. Carotenoid recovery and IL recycling was then achieved by using water as an antisolvent. The application of water led to the formation of a lipidic top phase that was rich in carotenoids, and an aqueous bottom phase containing the IL. This strategy enabled the almost complete recovery of the pigments from the IL solution. The recycled IL could be reused three times, even though the extraction efficiency was significantly reduced during the three cycles.

The summary of the studies listed in Table 2 and Table 3 points out the immense scientific interest on the extraction of phenolic compounds and carotenoids. These methods have been investigated for the recovery of other bioactive compounds such as polysaccharides and pectins, but to a lesser extent (Table 4). The results obtained so far indicate that UAE represents an efficient method to extract polysaccharides of higher purity when compared to the conventional extraction methods (e.g., hot water extraction, Soxhlet, etc.) [108]. Moreover, the extracts contained significant concentrations of β-glucans with a 0.26 degree of branching, suggesting high biological activity (ranging between 0.2 and 0.33). SWE provides high extraction yields of polysaccharides, however these recovery yields are reduced depending on the rigidity of the raw materials [60]. Pressurized CO_2_ extraction yielded fewer pectic oligosaccharides (3.8%) in comparison to the conventional method with HCl (9.9%), however it has a higher degree of esterification (DE) and a larger molecular weight was achieved [109]. Subcritical water was proven to selectively extract proteins with antioxidant activity from shellfish waste streams, after the proper temperature manipulation [110].

**Table 4:** Recent advances regarding the recovery/production of oligosaccharides and pectins from waste and by-product streams.

Raw Material	Method	Results	Reference
Corn stover	UAE (40 kHz, 360 W, 30 min) and hydrothermal pretreatment at 215 °C, for 1.5 h, followed by enzymatic hydrolysis with cellulase (15 FPU/g) with 2% solids at 50 °C	XOS yield: 80.40% (21.68% X2–X4, 58.72& X > 4)	[111]
*Caragana korshinskii*	Liquid hot water pretreatment, 160 °C, for 120 min with 4 g/L aqueous choline chloride and 1:20 solid-to-liquid ratio	XOS yield 36.59%	[112])
Food processing wastes	UAE at 300 W, ambient temperature	galactolipids	[113]
Finger citron pomace	EA (0.25 mg/mL pectinase, at 45 °C, pH 4.5, 2 h) and UAE (1:50 solid-to-liquid ratio, pH = 1, 90 °C for 1.5 h)	Pectic oligosaccharides yield of 64.5% with 94.07% DPPH antioxidant activity value	[114]
Satsuma mandarin orange peels	Pressurized CO_2_ at 90 °C for 90 min and at pressures of 1–3 MPa with 40% solids	Pectic oligosaccharides with a yield of 3.8% and 94% DE	[109]
Artichoke by-products	UAE (pulsed mode, 2 s on/1 s off, 30% amplitude) with 13.9% Celluclast 1.5 L, 6 h extraction time, 6.5% *w*/*v* solid-to-liquid ratio, 50 °C, under constant stirring condition at 200 rpm	10.9% pectin yield	[115]
Walnut processing waste	UAE at 200 W, for 10 min, at pH 1.5 with citric acid and 15 *v/w* liquid-to-solid ratio	Pectin extraction yield 12.78 ± 0.83%	[116]
Fenugreek seeds	High voltage atmospheric cold plasma with air at 80 kV for 30 min, followed by soaking at 5% NaCl, pH adjustment to 3 with acetic acid, 50 °C for 24 h, intermitted shaking at 250–300 rpm	122% increase in extraction yield of galactomannans	[117]

The number of studies proposing NADES as alternative green solvents for the extraction of a variety of bioactive compounds is constantly increasing (Table 5). NADES constitute attractive green solvents as they are composed of natural, non-toxic and biodegradable compounds. In addition, NADES that are synthesized by organic acids have shown antimicrobial properties, enhancing the overall biological effect of the extracts [106]. Although promising, more studies shall be implemented in the future to fully elucidate the potential hidden, toxic effects of NADES, especially for human health.

Cold plasma treatment comprises another emerging technology in the extraction of bioactive compounds. This process is currently employed in the food industry for its significant antimicrobial efficiency [90]. To date, the cold plasma treatment has been investigated for the extraction of phenolic compounds from grape [90] and tomato pomaces [91], and galactomannans from fenugreek seeds [117].

The recovery of organic acids follows different principles in comparison to the above described compounds, as they can be easily recovered by ion-exchange chromatography or via membrane separation [5,118]. Characteristic examples of such organic acids include citric and tartaric acids which are abundant in various fruit by-products. Tartaric acid is abundant in wine lees and it can be easily extracted via acidification and precipitation, and re-utilized in the food industry as a preservative [119]. Similarly, citric acid has been reported to be recovered from kiwi by-products and then absorbed again within the kiwi industry to reduce the browning of the fruit slices [5].

**Table 5:** Recent advances regarding the recovery/production of mixtures of bioactive compounds and proteins from waste and by-product streams.

Raw Material	Method	Results	Reference
***Mixture of bioactive compounds***			
*Capsicum annuum* industrial waste	CO_2_-SFE, at 40 °C and 250 bar with EOH as modifier	Extraction yield: 10.08% TPC: 15.77 ± 0.51 mg GAE/g extract; TFC: 0.60 ± 0.02 mg RE/g extract β-carotene: 1.26 ± 0.01 mg/g extract; lycopene: 0.49 ± 0.02 mg/g extract	[120]
Orange peel waste	NADES composed of choline chloride and ethylene glycol	D-limonene: 0.5 mg/g; Polyphenols: 45.7 mg/g; Proteins: 7.7 mg/g	[121]
Melon peels	DES (sodium acetate: urea: water at molar ratio 1:3:1.6), 90 °C for 10 min	Oligosaccharides: 8.84 g/100 g (90% oligogalacturonides); TPC: 4.35 mg GAE/g; Protein: 2.87 g/100 g; Pectin: 7.95 g/100 g (49.44% galacturonic acid content)	[122]
Tomato peel waste	HHPE for pectin recovery with nitric acid at 80 °C, 300 MPa system pressure, 1:20 solid-to-liquid ratio for 30 min, and UAE for polyphenols extraction with 70% EOH, 400 W, 30 kHz, 95% amplitude, 1:50 solid-to-liquid ratio and 15 min extraction time	Pectin extraction yield: 9.1%; Total phenolics: 1625.7 mg/100 g	[123]
Black mulberry pomace	MAE at 700 W, 300 s irradiation time, pH equal to 1.42 adjusted with citric acid and 20 mL/g liquid-to-solid ratio	10.95% pectin yield; 12.11% phenolics yield	[124]
Barley and canola straws	Subcritical water and pressurized 20% EOH at 180 °C, at 50 bar	TPC: 45.4 ± 1.8 mg GAE/g barley straw & 52.9 ± 2.0 mg GAE/g canola straw; Carbohydrates: 527.6 ± 0.5 mg GR/g barley straw and 442.7 ± 14.8 mg GE/g canola straw	[125]
***Protein***			
Kitchen wastes	MW and PHWE, 1:30 *w/w* solid to liquid ratio, 800 rpm stirring, 850 W, 160 °C	Protein: 8.27 ± 0.13 g/100 g extract; TPC: 1.34 ± 0.02 g/100 g; Trolox equivalent antioxidant capacity: 0.96 ± 0.07 g/100 g	[126]
Brewer’s spent grain (BSG),	MAE at 110 °C, 10 min extraction time, 0.5 M NaOH	Protein extraction: 14.6 kg/100 kg	[127]
Bamboo shoots (TBS) and processing wastes (basal bamboo shoot (BBS) and sheat)	NADES synthesized by choline chloride and levulinic acid (molar ratio 6), at 80 °C, 50 min extraction time, 40% water content, 30 mg/L and 70 mg/mL solid-to-liquid ratio for TBS and BBS	Protein extraction yields: TBS: 39.16 ± 1.22 mg/g; BBS: 15.46 ± 0.30 mg/g; Sheath: 9.54 ± 0.17 mg/g	[128]
Shellfish waste streams (brown carb shell residues)	SubWE at 200 °C at 1:15 solid-to-liquid ratio, 15 min	Protein: 8.5 g/100 dry residue	[110]

### 4.2. Enzymatic Recovery

Enzyme-assisted extraction (EAE) belongs also to the group of green technologies that are utilized for the recovery of bioactive compounds, usually in combination with one of the previously analyzed processes. The main principle behind EAE is to break the linkages between the compounds in question and the plant cell wall, facilitating their recovery [68]. The enzymes that are most commonly used belong to hydrolases such as cellulases, hemicellulases, pectinases, proteinases and ligninases. The nature and the characteristics of the raw materials define the enzymes that will be employed, the enzyme load, solid to liquid ratio and reaction time. Such characteristics include particle size, structure complexity and heterogeneity [129]. The enzymatic recovery of bioactive compounds is reckoned to be a safe process, as it is carried out under mild conditions (low temperatures and almost neutral pH values) and without toxic solvents, thus legally categorizing the derived products as “natural” [130]. The main drawbacks that are limiting the commercialization of EAE is the high cost of enzyme production, the difficulty to recycle and reuse the enzymes, as well as the preparation of tailor-made enzymes to achieve high efficiencies, depending on the raw material. An alternative to mitigate the cost of production is the utilization of inexpensive substrates. For instance, the production of crude proteases from palm oil by-products [131] and crude enzyme consortia from mixtures of milling by-products and confectionary side-streams [132] has already been demonstrated. Scaling-up EAE is also challenging, as enzymatic activity, and thus the efficiency, is influenced (among other factors) by the solids concentration, the dissolved oxygen, the temperature, and the mass transfer phenomena [60].

Enzymatic treatment will rupture the plant cell wall at specific regions, releasing bioactive compounds with a higher selectivity in comparison to those acquired by the use of other green methods [129]. Especially in the case of phenolic compounds, which can be either bound to proteins or found in glucoside forms, enzymes can increase their release in the extract. The selection of the adequate enzyme or the enzymes’ combination is crucial for the process’ optimization as well as for establishing the suitable parameters that will govern the enzyme kinetics and activity at the given substrate [129]. However, EAE shows low extraction yields when implemented as a standalone method. On the other hand, methods such as SFE and UAE are not effective at easily disrupting the multiple plant membrane layers, limiting their efficiency only on the free forms of bioactive compounds [133]. The combined extractions of EAE with another green technology method would enhance the extraction efficiency as well as the selectivity of the bioactive(s) that will be recovered. The most common combinations with EAE found in the literature mainly include UAE and MAE. For example, UA in combination with 3.3% Alcalase enhanced almost 30% the TPC from waste mango peels when compared to the sole use of UAE [134]. Enzyme-treated blackcurrant press cake (derived after the extraction of the juice) led to a higher anthocyanin recovery, while the extracts showed antiviral activities [135].

Even though most studies are focusing on phenolic compounds and other antioxidants (carotenoids) extraction, enzymes could also be deployed for the production of bioactive peptides. Tacias-Pascacio et al. [136] presented a very comprehensive review regarding the application of the commercial enzyme Alcalase, especially for the production of bioactive peptides from diverse protein sources.

The enzymatic recovery of polysaccharides has also been demonstrated, as by using this process, the three-dimensional structure of polysaccharides can remain intact, minimizing the possibility of bioactivity loss [60]. The production of oligosaccharides with prebiotic potential after an enzymatic treatment (hydrolysis) has been demonstrated, also, on the industrial scale [37]. Pectic oligosaccharide fractions, with improved characteristics in terms of their physicochemical and functional properties, were obtained by an ultra-high pressure enzyme extraction process from red pitaya peels [55]. In this method, the enzymatic hydrolysis (with a mixture of cellulase, pectinase and papain) occurred inside a high-density polyethylene bag, at temperatures ranging from 40–60 °C and pressures of 200–400 MPa. This process enhanced the recovery of pectic polysaccharides by more than 90% in comparison to that of conventional HRE methods. Wu et al. [57] have shown that the extraction method can cause variations in both the structure and the carbohydrate content of pectins that are extracted from raspberries. Comparing pectins obtained from either hot-acid or EAE methods, the latter was proven to be composed of more arabinan sidechains, hence demonstrating higher immunomodulatory properties.

The agar production industry results in a solid residue which accounts for more than the 2/3 of the initial raw material (the red algae *Celidium sesquipedale*). This residue is currently underexploited, although it comprises a source of proteins (containing all the essential amino acids), carbohydrates, and phenolic compounds. The enzymatic hydrolysis of this industrial residue increased the TPC of the resulting extract as well as the amount of proteins and carbohydrates [137]. Cellulase was the most efficient enzyme for the release of phenolic compounds and sugars, whereas the addition of protease was indispensable for protein extraction. Polysaccharide fractions from *Pleurotus eryngii* residues attained after an enzymatic treatment with snailase (mixture of 20–30 enzymes, mainly cellulase, pectinase and protease), demonstrated potential anti-ageing properties on various organs [138]. The commercial enzymatic preparation of Cellic CTec3 catalyzed the production of fucosylated human milk oligosaccharides from depectinized citrus residues [139]. Evidently, enzymes offer several advantages and significant potential for the sustainable extraction of bioactive compounds.

### 4.3. Microbial Production

**Figure 3:**
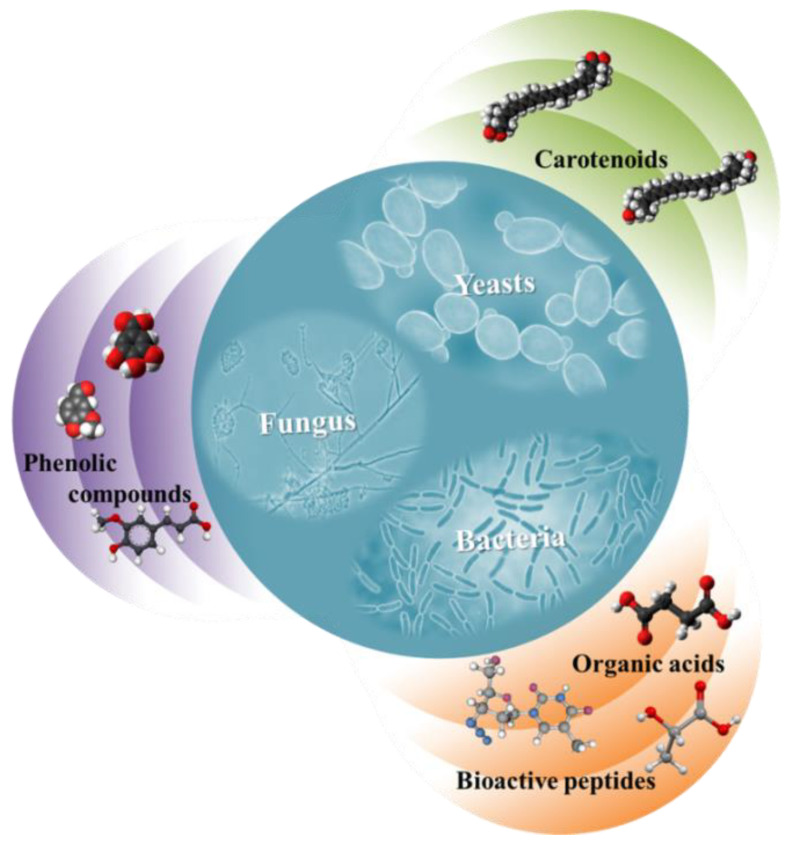
Exploitation of microbial cells for the sustainable production of bioactive compounds.

The biotechnological production of bioactive compounds is another alternative instead to the extraction from plants and residues. Agro-industrial residues are rich in fermentable sugars that could be converted into microbial products, either directly or after hydrolysis. Many microorganisms are able to synthesize metabolites of interest, i.e., those exhibiting a specific bioactivity (Figure 3). The exploitation of inexpensive by-products or waste streams as raw materials for microbial fermentations is a process that is in compliance with the new era of bioeconomy and renders the biotechnological production of bioactive compounds as a very attractive subject of research.

Many research groups have focused on the production of organic acids from renewable resources. Table 6 summarizes some characteristic examples of studies on the microbial production of various organic acids. López-Gómez et al. [140] summarized the most important studies of the last decade regarding the production of L-lactic acid from renewable resources in continuous fermentations. According to this review, inexpensive resources such as tapioca starch hydrolysate, molasses, whey, lignocellulosic hydrolysates, defatted rice bran hydrolysate, sludge from anaerobic digestion and corn stover hydrolysate have been successfully utilized as fermentation substrates for lactic acid production. The fermentable production of D-lactic acid from the food industry’s by-products (e.g., coffee mucilage, rice husks and acid whey) has also been evaluated [141].

Other organic acids with many applications in the chemical, pharmaceutical, plastic and food industry such as succinic acid and fumaric acid can also be formulated from renewable resources via biotechnological routes. The biotechnological production of succinic acid has also been progressed to its industrial implementation by companies such as Myriant, Reverdia and Succinity. Wild-type strains including *Actinobacillus succinogenes*, *Anaerobiospirillum succiniciproducens* and *Mannheimia succiniciproducens* [156] as well as yeast strains such as the metabolically engineered *Saccharomyces cerevisiae* and *Yarrowia lipolytica* have been evaluated for succinic acid production [157]. Some examples of succinic acid production from inexpensive resources encompass the utilization of sugar beet pulp hydrolysates [158], spent sulphite liquor [159] and grape pomace and stalks [160]. Su et al. [161] demonstrated the exploitation of *Panax ginseng* residues for the co-production of multiple functional components. Physico-chemical separation was initially applied for polysaccharide and ginsenoside recovery. The resulting residue was then enzymatically hydrolyzed and converted to succinic acid by *Escherichia coli* ZW333.

Raveschot et al. [162] describe the ability of some species of lactic acid bacteria (LAB) to hydrolyze proteins in order to use them as a nitrogen source for their growth. In this way, the simultaneous production of peptides is achieved, while some of them belong to the category of bioactive peptides. The produced bioactive peptides can be used for the functionalization of dietary products (mostly milk products), or they can be extracted for other applications.

Carotenoids production using microorganisms has also been proposed in the literature as a sustainable alternative to their extraction from plants or their chemical synthesis. Yeasts, bacteria and algae are capable of producing different carotenoids via the assimilation of a plethora of inexpensive substrates such as cooking oil waste, molasses, waste hydrolysates and glycerol [54].

## 5. Trends in the Application of Sustainable Functional Compounds toward the Development of Clean Label Foods

The term “clean label food” has emerged to describe food products that are not only free of artificial compounds, but are also produced in a sustainable manner [163]. As they are not yet legally defined, clean label foods are open to different interpretations, yet it is generally acknowledged that a “clean” label product contains natural ingredients and does not include over-processed compounds. In view of the new EU directives and sustainability goals, it would be quite strange to not allocate “sustainable production” into this relatively “broad” definition.

The concept of “clean” label foods includes products that are free of ingredients that consumers will find unacceptable. Synthetic additives, preservatives, artificial colors, saturated fatty acids and hydrogenated lipids constitute some of these components. Therefore, a major turn toward natural ingredients, fewer additives and more advanced health benefits has emerged along with significant challenges for the food manufacturers. The inclusion of bioactive compounds that are listed in this review in novel food formulations could pave the way for more sustainable and clean label food products.

### 5.1. Applications as Food Additives and Preservatives

Organic acids are traditionally used as food preservatives due to their antimicrobial activity. Quattrini et al. [164] observed the synergistic effects of the addition of acetic acid together with propionate and sorbate on reducing the fungal spoilage of bread. To this end, the authors proposed *Lactobacillus hammesii* for sourdough fermentation and acetate formation as an efficient manner to reduce the propionate concentration in bread.

Kiwi by-products are a source of bioactive ingredients such as phenolic compounds, enzymes (actinidin) and pectins that have been efficiently employed as natural preservatives in food products [5]. Phenolic extracts from kiwi by-products were added in beef meat and camel’s milk, stabilizing its texture and preventing discoloration, while maintaining the product’s sensorial characteristics [5]. Cheese manufactured with kiwi extracts was rich is polyphenols and phytosterols, while kiwi protein could replace soy protein. Extracts obtained from figs and olive leaves were tested individually and combined as milk preservatives, increasing its shelf life to 11 days [165]. Tsouko et al. [166] incorporated phenolic-rich extracts from palm oil by-products in sunflower oil. The crude extract significantly increased the oil’s stability against oxidation.

The effective replacement of health hazardous compounds that are currently utilized by the meat industry (e.g., nitrites, phosphates, etc.) has been on the center of attention for various studies, and has also been recently reviewed [167]. Polyphenolic-rich extracts, especially those from fruit and vegetable by-products, constitute adequate candidates to prevent the lipid and protein oxidation of meat products, and at the same time, they inhibit microbial spoilage. To this end, Tamkutė et al. [168] evaluated the protective effects of extracts derived from cranberry pomace against foodborne pathogens of pork-meat burgers, ham and slurry. The extract successfully inhibited *Listeria monocytogenes* growth as well as the formation of oxidation products, signifying the potential of these kinds of extracts as natural food preservatives.

The addition of cranberry pomace (CP) in the mixture of a dry sausage preparation reduced the risk of *Salmonella* spoilage, without significantly affecting the sensorial properties of the final product [169]. The antimicrobial properties of CP were attributed to the high content of bioactive phenolic compounds, concluding that this by-product could be valorized as a whole in functional foods preparation.

Phosphates are added into meat products to increase their water-holding capacity and to suppress lipid oxidation. Food industry by-products that are rich in crude fibers (e.g., fruit peels) have been suggested as replacers of phosphates [167]. The addition of avocado peel extract (TPC equal to 281.4 mg GAE/g extract) in beef and soy burgers was proven to prevent or decrease their oxidative effects and the production of toxic compounds (e.g., acrylamide, heterocyclic aromatic amines) during cooking [170]. The protective effect of the extract was compared, and was found to be superior to the addition of sodium ascorbate to the burgers. The extract was obtained by a UAE method with the use of ethanol and water, meaning that the process was free of hazardous solvents.

Astaxanthin extracted from shrimps was encapsulated in a complex of whey protein isolate and Persian gum, and was utilized as a natural colorant in a simulated beverage [171]. Encapsulation enhanced the color stability however the carotenoid’s concentration was reduced over time.

### 5.2. Applications as Health-Promoting Agents

Carotenoids serve, not only as natural pigments providing attractive colorations to foodstuffs but can also induce multiple health benefits. The microbial production of carotenoids is flourishing in Asia, where many marketable products (e.g., cheese, wine, fish and meat products) include microbial carotenoids as active ingredients and colorants [54]. Besides the microbial counterparts, carotenoids that are extracted from food by-products have been investigated for the production of novel foodstuffs. Šeregelj et al. [172] integrated carrot waste extracts rich in carotenoids into yogurt, after encapsulation by electrostatic extrusion. The antioxidant activity of the resulting fortified yogurt was enhanced, while its physico-chemical properties were not compromised. Recently, Tiwari et al. [173] prepared a freeze-dried powder derived from a stabilized emulsion composed of carotenoids (recovered from carrot pomace) and flaxseed oil. The powder was subsequently used for the formulation of flavored milk. The authors concluded that the emulsion increased the total carotenoid content, antioxidant activity and the fat and protein contents of the final products and thus, it can be considered a functional natural food colorant.

Cookies fortified with phenolic compounds from pomegranate peel were prepared in the study of Kaderides et al. [174]. In order to minimize the losses during baking, the extracts were encapsulated in orange juice by-products. Encapsulation had a positive impact on the preservation of the phenolic compounds, however the authors reported that their concentration was still highly influenced by the baking temperatures. Pomegranate peel extracts were incorporated into sponge cake aiming to reduce its glycemic index (GI) [175]. In vitro tests demonstrated an inhibitory effect of the extracts on α-amylase and α-glucosidase activity, reducing starch digestibility and GI by more than 40%. Texture and color were affected by the extract, however, the addition of it of up to 1% was positive in terms of consumers’ acceptance.

In another study, pomegranate rind powder was applied as a substitute for wheat flour for the preparation of functional cookies [176]. The novel cookies contained higher amounts of phenolic compounds and had increased antioxidant activities when compared to the control samples. However, the study did not provide any information of the possible organoleptic effects of the pomegranate rind powder.

As shown in Table 2, marine-based waste streams merit exceptional sources of bioactive compounds. Fish collagen bioactive peptides have been established as versatile molecules, finding many food applications. Consequently Ayati et al. [177] prepared a yogurt enriched with bioactive peptides obtained from fish collagen. The organoleptic characteristics of the fortified yogurt were not altered. The antioxidant activity was enhanced, and it also presented ACE and DPP-IV-inhibitory characteristics, suggesting its potential for controlling blood pressure as well as possible anti-diabetic properties. Soluble dietary fibers derived from grapefruit peels after an MWAE-UAE process were integrated in wheat bread for the formulation of a functional product [178]. The sensory qualities of the final product were acceptable, while the addition of dietary fibers decreased the release rate of glucose under the in vitro digestion tests.

Collagen-derived peptides were co-fermented with jackfruit juice for the preparation of a novel functional beverage in the work that was conducted by Ma et al. [179]. The juice was provided to immunosuppressed mice, aiding their immune’s system response. Moreover, the juice supply presented a positive effect on gut microbiota, increasing the production of short-chain fatty acids.

In addition, plenty of studies have reported the application of various oligosaccharides as prebiotics in functional foods. Likewise, in the study of Hesam et al. [180], XOS were initially generated from sugarcane bagasse under alkaline conditions and enzymatic hydrolysis. Then, their prebiotic properties on the viability of the probiotic strain *Bifidobacterium animalis* Bb-12 were evaluated in a synbiotic pomegranate juice preparation. The cell viability displayed an increase, even after one month at 4 °C. In the same context, guava purée by-products (seed and pulp) were enzymatically hydrolyzed with a commercial xylanase and cellulase and further utilized in a functional yogurt preparation. The prebiotic effect of the enzymatically induced products was evidenced by a 77.6% increase in the starter culture’s CFU per mL, while other parameters such as adhesiveness and stickiness were also enhanced.

### 5.3. Case Studies of Clean Label Products Using Sustainable Functional Compounds

**Figure 4:**
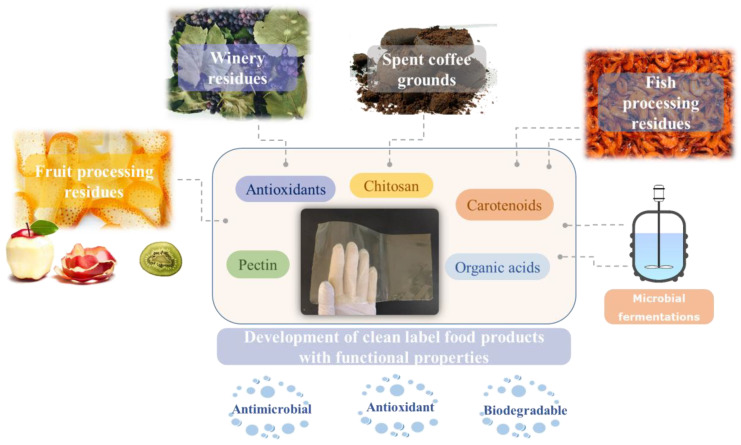
Valorizing agro-industrial waste streams via extraction of bioactive compounds or biotransformation: a holistic approach for the production of clean label foods products.

The application of bioactive compounds into active packaging comprises another valorization strategy of the residue-derived extracts. Moreover, the development of bio-edible and/or biodegradable food packaging is also imposed by the general sustainability directives. In this context, many studies have been focusing on the manufacture of such sustainable food packaging, using bio-based materials (Figure 4).

Recently, Papadaki et al. [181] elaborated a novel valorization scheme of whey protein through the development of novel edible films. Phenolic compounds extracted from spent coffee grounds were also included in the films, resulting in enhanced antioxidant activities, whereas the mechanical properties of the films were also positively affected. The inclusion of mushroom biomass was also reported to improve the antioxidant activity of whey protein films, while reducing their water vapor permeability [182].

Chitosan films enriched with carotenoids extracted with ILs were developed by De Souza Mesquita et al. [58]. The resulting films could be employed as alternative packaging materials in the food industry. The carotenoid-enriched films presented better properties in comparison to the control ones in terms of their elasticity, weight loss during storage, and antioxidant activity, even though the tensile strength of the films was decreased.

Kiwi pectins were incorporated into biodegradable films for chicken wrapping, resulting in its preservation for up to 9 days, with low lipid oxidation [5]. Çavdaroğlu et al. [183] prepared an active edible coating from a fig-derived pectin. The novel coating exhibited an antimicrobial activity and was applied on fresh melon slices. Linn et al. [184] formulated novel biodegradable films utilizing pectin extracted from the leaves of *Cissampelos pareira*. The pectin was blended with polyvinyl alcohol (PVA) and crosslinked with 16% succinic acid. The membranes were tested on food counterfeits exhibiting antioxidant activity. Their biodegradability was also confirmed by a soil burial test of almost one month. It should be noted that the addition of bioactive compounds from fruit pulps into edible films is legally accepted by the FDA (Food and Drug Administration, Silver Spring, MD, USA) [58].

The addition of organic acids in the structure of biodegradable films serves multiple purposes such as exhibiting antimicrobial activity, while at the same time acting as plasticizers and/or as crosslinking agents, thus rendering the film more elastic. For example, citric acid was added into biodegradable films, composed of polyvinyl alcohol (PVA) and carboxymethyl chitosan (CMCS), as both an antimicrobial agent and a crosslinker [185]. Citric acid increased the thermal stability of the films and reduced the water losses that were experienced when used on cherry tomatoes and strawberries. Moreover, the shelf life of the fruits was extended, indicating its potential as a food packaging material. Likewise, the mechanical and antimicrobial properties of potato starch/chitosan films were enhanced when 15% citric acid was incorporated into their structure [186]. Succinic acid is another organic acid that presents positive effects as a crosslinking agent. More specifically, Gabriele et al. [187] developed chitosan-succinic acid-based films neutralized by NaOH. The resulting films demonstrated improved physicochemical properties when compared to the single chitosan membranes, due to the formation of amide bonds and ionic crosslinking.

## 6. Conclusions and Future Perspectives

This review elaborated on the state-of-the-art trends in producing and recovering functional compounds for the development of sustainable food systems. The large number of publications validates the interest of the scientific community toward the efficient production of these compounds, which is also directed by the new EU regulations along with the consumers’ demand for healthier products. The results that have been obtained so far are promising and novel, whereas functional and clean label food products are being constantly developed and further optimized. Nonetheless, it is unequivocal that more remains to be done before establishing the development of sustainable and clean label food systems. Bioeconomy might, currently, be a global tendency, but governments and societies are obliged to start acting in a dynamic manner within its pillars and accelerate the transition to circular economy and sustainability.

For instance, even though there are plenty of studies in the literature reporting on the application of naturally resourced, derived oligosaccharides (XOS, FOS, GOS, etc.) as prebiotics in functional foods, many research cases still depend on specific commercialized oligosaccharides [35,188], as in the case of the study by Bis Souza et al. [189], where FOS under the commercial name NutraFlora^®^ P95 have been evaluated as fat replacers in sausages. The lack of studies dealing with the effects of such compounds on humans is the main reason behind this. Different disciplines should collaborate in a multi-targeted approach in order to completely unravel the potential of these components and identify their benefits or side effects in the human organism.

An impediment on the global use of such compounds correlates with their chemical, physical or enzymatic instability and concentration fluctuations from batch to batch, thus more research should be conducted to overcome these drawbacks. Besides the organoleptic aspects, there are still plenty of technological aspects that need to be assessed. The majority of the emerging extraction and production techniques are still centered on low TRL, and their cost is too high to counterbalance. Apart from dealing with the techno-economical obstacles, the consumers’ acceptance is another aspect that needs to be addressed. Food legislation should be appropriately modified to include clean label products. Sustainably produced foods should be properly certified and labelled. Likewise, public awareness campaigns should promote and increase the visibility of clean label products, highlighting both their health and environmental benefits. Undoubtedly, the transition toward sustainable food systems requires adequate financial support and training to assist the agri-food sector to comply with the demands of the new era. Appropriate legislation, multi-stakeholder forums and even probably some other forms of motivation (e.g., tax reduction) are some proposals that could encourage the agri-food industry to conform.

Evidently, in compliance with the European Commission’s “Farm to Fork” Strategy, the member states are encouraged to promote the transition to sustainable food systems. This strategy mainly targets to developing products with zero environmental impact, abating climate change and increasing the access to nutritious, affordable and sustainable food. The adoption of efficient measures elicits the only tool to take bioeconomy to the next level: its actual implementation.

## Figures and Tables

**Table 1:** Summary of the novel technologies employed for the green recovery of functional compounds.

Method	Principle	Advantages	Disadvantages	Target Compounds	Reference
Subcritical water extraction (SWE)	Use of water as extraction solvent in liquid state under high pressure at a temperature between 100 and 374 °C	Extraction of less-polar compounds with non-toxic solvent (water)	At temperatures above 240 °C, most of bioactive compounds appeared to be unstable and can be destroyed	Polysaccharides, phenolics	[60,61]
Sub- and supercritical fluid extraction (SFE)	Use of gases in their sub- or supercritical fluid state	Using CO_2_: non-toxic, inexpensive, non-flammable, modest critical point, easily recovered, suitable for heat-sensitive compounds, no product degradation due to absence of air and light, low energy consumption	Using CO_2_: requires a co-solvent (e.g., EOH, water) for the efficient extraction of polar compounds, high capital cost, requires technical expertise	Polyphenols, carotenoids, lipids, essential oils	[62,63,64]
Pulsed electric fields (PEF)	Cell membrane rupture due to electrical pulses (0.1–100 kV/cm) with a duration of 100–1000 μs	Extracts of higher purity, selectivity, low energy consumption, short processing time, suitable for temperature-sensitive compounds	Costly equipment maintenance, control parameters optimization	Polyphenols, proteins, polysaccharides	[50,60,63,65]
Microwave assisted extraction (MAE)	Application of microwave energy at frequencies of 300 MHz–300 GHz causing dipole rotation or ionic conduction induced by microwave energy	Low solvent requirements or solvent-free extraction, easy set-up, low economic and environmental impacts	Higher effectiveness for polar compounds; non-selective, unsuitable for heat-sensitive compounds, non-homogenous heat distribution, high energy consumption	Polyphenols, pectins, polysaccharides, bioactive peptides	[63,65]
Ultrasound assisted extraction (UAE)	Acoustic cavitation generated by ultrasonic waves (20 kHz–10 MHz)	Low solvent requirements, easy set-up, low economic and environmental impacts	No selectivity; long extraction times depending on target compound; uncontrollable energy transfer	Phenolic compounds, polysaccharides, proteins, lipids, pigments	[66]
Pressurized liquid extraction (PLE)	Application of high pressure (3–20 MPa) and temperature (50–200 °C); solvent temperature above boiling point	Rapid extraction time/rate, solvent volume reduction, high recovery yields, use of safe solvents (e.g., water), simple equipment	High cost of equipment, unsuitable for temperature-sensitive compounds	Flavonoids (especially anthocyanins), sugars, carotenoids	[4,65,67]
Pulsed ohmic heating extraction (POHE)	Ohming heating in conjunction to electric field (E < 100 V/cm) (conversion of electrical energy into heat energy)	Fast extraction	Requires process design to achieve homogenous sample heating; Quality and safety procedures for commercial use	Polyphenols, bioactive peptides	[4]
High hydrostatic pressure extraction (HHPE)	Application of high pressure (300–1000 MPa) at room temperature for 3–5 min	Faster extraction times, higher recovery yields and efficiencies, low energy consumption, applicable for polar and non-polar compounds, suitable of heat-labile compounds	Expensive equipment, maintenance, well-trained workforce due to high pressures employed	Carotenoids, pectins, phenolic compounds	[4,50]
Cold plasma assisted extraction	Application of cool ionized gas-containing ions, electrons, reactive neutral radicals and ultraviolet photons that rupture plant cell walls	Non-thermal process suitable for thermos-labile compounds, operation at atmospheric pressures, inexpensive equipment, low energy consumption	Available data only in lab-scale experiments, requires process optimization and study on possible alterations in the structure of the compounds	Phenolic compounds, protein, polysaccharides	[4,67]
Ionic liquids	Organic salts that are composed of organic cations and organic/inorganic anions—used as green solvents or used alone or in combination with other techniques	Low volatility, wide range of polarity	Expensive, possibly toxic, flammable, low stability	Phenolic compounds, carotenoids, oligosaccharides	[68]
Natural deep eutectic solvents	Used as green solvents or used alone or in combination with other techniques	Low volatility, wide range of polarity, low cost, easy preparation, stability, “tenability”	Short life, pending evaluation of potential toxicity	Polar and non-polar compounds	[68]

**Table 2:** Recent advances in the extraction of phenolic compounds from agro-food residues and waste streams.

Raw Material	Method	Results	Reference
*Citrus depressa* Hayata peels from ripe and urnripe fruits	UAE with 50% EOH at solid-to-solvent ratio 1:100, ambient temperature and 50 min (unripe) or 40 min (ripe)	Unripe fruit peels: Rutin: 6.265 mg/g; Nobiletin: 12.511 mg/g; Tangeretin: 7.016 mg/g. Ripe fruit peels: Rutin: 3.033 mg/g; Nobiletin: 7.439 mg/g; Tangeretin: 3.569 mg/g.	[70])
Dried onion skins	PEF (as pretreatment step) at 2.5 kV/cm for 15 s and SWE at 145 °C for 15 min	19.25 ± 0.77 mg total quercetin per g (33.22% improved extraction yield in comparison to samples without PEF treatment)	[71])
Green asparagus roots	ILs: 0.5% 1-butyl-3-methylimidazolium chloride, at 1:10 S:L ratio, for 4 min; PEF: 1.6 kV/cm, 200 Hz, 20 μs pulse width	TFC with ILs: 122 mg RE/mL; TFC after PEF: 1.52–1.74 mg RE/mL	[72])
Pomegranate peel waste	SFE using CO_2_ and EOH as co-solvent at 20 MPa and 40 °C; UAE with EOH	SFE:11,561.84 ± 490.69 μg/g total polyphenols; UAE: 6940.74 ± 264.32 μg/g total polyphenols	[73]
Waste mango peels	UA and NADES (lactic acid:glycerol 5:1), 1:30 solid-to-liquid ratio, 30 min, 30 °C	TPC: 69.85 mg GAE/g; TFC: 16.5 mg QE/g	[74]
Turmeric residues	DES (choline chloride: propylene glycol, at 1:2 ratio) with 20% water, 1:40 solid-to-liquid ratio, 50 °C, 60 min	Curcumin extraction yield: 54.2 mg/g	[75]
Pomegranate peel waste	NADES composed of choline chloride: lactic acid (35:100), 1:20 waste-to-solvent ratio, 45 °C, 25 min extraction time	TPC: 4.14 mg GAE/mL	[76]
Onion skin	NADES composed of glycolic acid and L-proline with 30% w/w water, 50 mg solids with 1.5 g NADES, 30 min extraction time, 50 °C temperature	Quercetin: 18.56 ± 0.25 μg/mL	[77]
Uvaia residue	UAE with 40% amplitude, water as solvent, and 40 °C for 2.5 min followed by concentration of the extract with reverse osmosis	TPC: 332.22 mg GAE/100 g; TFC: 1300.18 mg/100 g	[78]
Cranberry pomace	PLE with ethanol 30–100%, at 40–160 °C, 50 or 200 bar	Max anthocyanins extraction (6.02–8.42 mg Cy3GE d.w.) with 100% EOH; Max TPC (84.96 ± 7.82 mg GAE d.w.) with 30% EOH at 140 °C, and 50 bar	[69])
Acerola and umbu residues	UAE (40 kHZ, 40 °C, 30 min) or simple shaking (120 rpm, 40 °C, 30 min) with 80% acetone	TPC acerola: 444.05 mg GAE/100 g; TPC umbu: 404.36 mg GAE/100 g	[79]
Custard apple leaves	PEF at 6 kV/cm with 70% EOH	TPC: 235.55 mg GAE/g dry extract	[80]
Mango seed kernels	CO_2_-SFE with 15% EOH, 11 MPa at 60 °C	TPC: 57.3 mg GAE/g extract; TFC: 13.6 mg QE/g extract	[81]
Chestnut shells	MW (2.45 GHz) and NADES (choline chloride: oxalic acid, 1:1), 0.5:5 waste-to-solvent ratio, 85 °C, 60 min extraction time	TPC: 295.2 ± 3.2 mg GAE/g dry waste	[82]
*Camelia sinensis* branches	MAE at 140 °C, 1:15 solid-to-liquid ratio	TPC: ~55 mg GAE/g	[83]
Rosemary leaves	PLE at 183 °C, 130 bar for 3 min	Rosmarinic acid: 10 ± 1 mg/g; carnosic acid: 21 ± 1 mg/g	[84]
Olive pomace	UAE (10 min) with β-cyclodextrin aqueous solutions, under stirring for 21 h, at 60 °C	TPC ~3 mg GAE/g	[85]
Ripe mango peels	UAE (436.45 W, 19.6 min and 59.8 mL/g liquid-to-solid) with NADES composed of lactic acid/sodium acetate/water (3:1:4)	TPC: 56.17 mg GAE/g dry waste	[86]
Saffron petals	OHAE at 225 V for 45 min, with 5% petals and 0.3 g NaCl	TPC: 928 mg/100 g; TFC: 238 mg/100 g	[87]
*Vitis vinifera* cv *Tannat* grape pomace	UAE at 100 W, at 30 °C, 50 min extraction time	TPC: 21.6 ± 3.8 mg GAE/g	[88]
Sea bream and sea bass residues	PEF at 7000 V and 10 Hz, 20 μs pulse width and 100 number of pulses, with dH_2_O at 1:1 liquid-to-solid ratio	Antioxidants with DPPH activity ranging from 33.8–71.8% depending on the residue	[89]
Grape pomace	High voltage atmospheric cold plasma treatment at 60 kV for 15 min, followed by extraction with 50% EOH at solvent at 1:25 solid-to-liquid ratio for 2 h, under stirring at 150 rpm	TPC enhanced by 22.8% and DPPH by 34.7%	[90]
Tomato pomace	High voltage atmospheric cold plasma treatment with nitrogen gas at 60 kV for 15 min, followed by extraction with 50% EOH at solvent at 1:40 solid-to-liquid ratio for 15 min, under stirring at 150 rpm	TPC: 1.033 ± 0.020 mg GAE/g (9.8% improvement)	[91]

TPC: total phenolic content; Cy3GE: cyaniding-3-glucoside equivalents; GAE: gallic acid equivalents; GE: glucose equivalents; TFC: total flavonoid content; RE: rutin equivalents.

**Table 6:** Characteristic examples of the microbial production of different bioactive compounds.

Compound	Fermentation Substrate	Conditions	Results	References
Lactic acid	Poplar hydrolysate; Corn stover hydrolysate	*L. brevis* ATCC 367 and *L. plantarum* ATCC 2102, Enzymatic hydrolysis of corn stover	31.8 g/L from poplar hydrolysate with yield 0.80 g/g and productivity 0.48 g/L/h; 31.2 g/L from corn stover hydrolysate with yield 0.78 g/g and productivity 0.43 g/L/h	[142]
	Cassava bagasse (CB)	*Lactobacillus amylovorus* (ATCC-33620) and *Lactobacillus acidophilus* (ATCC-4356), Enzymatic hydrolysis of CB, Corn Steep Liquor as nutrient and nitrogen source, No pH control	31.6 g/L productivity, 0.11 g/L/h	[143]
	Crust bread waste (CBW) hydrolysate and lucerne green juice	*Bacillus coagulans*, Enzymatic hydrolysis of CBW, Continuous fermentation	55 g/L yield, 0.48 g/g productivity, 11.28 g/L/h	[144]
	Defatted rice bran (DRB) hydrolysates	*Bacillus coagulans* A107, Enzymatic hydrolysis of DRB, Batch fermentations	75.9 g/L yield, 0.90 g/g productivity, 2.7 g/L/h	[145]
	Sweet sorghum juice	*Bacillus coagulans,* Pilot scale fermentation (50 L)	73 g/L lactic acid yield, 0.70 g/g productivity, 1.47 g/L/h	[146]
Succinic acid	Rice husk hydrolysate	Acid hydrolysis of rice husks at pressurized reactor with 2.2% (*v*/*v*) HCl at 174 °C (59 bar) for 46 min	12.5 g/L succinic acid yield 59.9%	[147]
	Glycerol	Metabolic engineered *Y. lipolytica* based on the strain ATCC MYA-2613 no pH control	110.7 g/L succinic acid yield 0.53 g/g	[148]
	Fruit and vegetable wastes hydrolyzed	*A. succinogenes* NJ113, Enzymatic hydrolysis of fruit and vegetable wastes with Solid State Fermentation with *Aspergillus niger* and *Rhizopus oryzae*	27.03 g/L succinic acid yield, 1.18 g SA/g sugar productivity, 1.28 g/L/h	[149]
Fumaric acid	Very high polarity (VHP) sugar and molasses from sugarcane mills	*Rhizopus arrhizus* NRRL 2582, 200 mg/L FAN from soybean cake hydrolysate	40 g/L fumaric acid yield, 0.86 g/g of total consumed sugars	[150]
	Brewery wastewater	*Rhizopus oryzae* 1526 immobilized mycelia on muslin cloth	43.67 g/L fumaric acid productivity, 1.21 g/L/h	[151]
Carotenoids	Cane molasses hydrolysate	Wild-type *R. toruloides* ACCC 20,341 and mutant *R. toruloides* M18	12.32 mg/L torularhodin and 5.21 mg/L torulene	[152]
	Wheat straw hydrolysate	*R. toruloides* NRRL Y-1091, Decolorization of the cellulosic hydrolysate with activated charcoal	24.58 mg/L carotenoids, yield 0.32 mg/g, productivity 0.26 mg/L/h	[153]
	Sugar cane molasses	*Rhodotorula mucilaginosa* CCT 7688, Corn Steep Liquor as nutrient and nitrogen source, Fed-batch fermentation	3726 μg/L total carotenoides	[154]
Bioactive peptides	Soy milk	*Lactobacillus plantarum* C2, Fermentation of soy milk, Analysis with LC-MS/MS	Detection of 17 biofunctional peptides with antioxidant and ACE-inhibitory activities	[155]

## Data Availability

No new data were created or analyzed in this study. Data sharing is not applicable to this article.
